# Active subfractions of *Abelmoschus esculentus* substantially prevent free fatty acid-induced β cell apoptosis *via* inhibiting dipeptidyl peptidase-4

**DOI:** 10.1371/journal.pone.0180285

**Published:** 2017-07-17

**Authors:** Chien-Ning Huang, Chau-Jong Wang, Yi-Ju Lee, Chiung-Huei Peng

**Affiliations:** 1 Department of Internal Medicine, Chung-Shan Medical University Hospital, Taichung, Taiwan; 2 Institute of Medicine, Chung-Shan Medical University, Taichung, Taiwan; 3 Institute of Biochemistry, Microbiology and Immunology, Chung-Shan Medical University, Taichung, Taiwan; 4 Department of Pathology, Chung-Shan Medical University Hospital, Taichung, Taiwan; 5 Division of Basic Medical Science, Hungkuang University, Shalu District, Taichung, Taiwan; Institute of Biochemistry and Biotechnology, TAIWAN

## Abstract

Lipotoxicity plays an important role in exacerbating type 2 diabetes mellitus (T2DM) and leads to apoptosis of β cells. Recently dipeptidyl peptidase-4 (DPP-4) inhibitors have emerged as a useful tool in the treatment of T2DM. DPP-4 degrades type 1 glucagon-like peptide (GLP-1), and GLP-1 receptor (GLP-1R) signaling has been shown to protect β cells by modulating AMPK/mTOR, PI3K, and Bax. The anti-hyperglycemic effect *of Abelmoschus esculentus* (AE) is well known, however its mucilage makes it difficult to further examine this effect. In our recent report, a sequence of extraction steps was used to obtain a series of subfractions from AE, each with its own composition and property. Among them F1 (rich in quercetin glucosides and pentacyclic triterpene ester) and F2 (containing large amounts of carbohydrates and polysaccharides) were found to be especially effective in attenuating DPP-4 signaling, and to have the potential to counter diabetic nephropathy. Hence, the aim of the present study was to investigate whether AE subfractions can prevent the palmitate-induced apoptosis of β cells, and the putative signals involved. We demonstrated that AE, and especially 1 μg/mL of F2, decreased palmitate-induced apoptosis analyzed by flow cytometry. The result of western blot revealed that palmitate-induced decrease in GLP-1R and increase in DPP-4 were restored by F1 and F2. The DPP-4 inhibitor linagliptin decreased the expression of caspase 3, suggesting that DPP-4 is critically involved in apoptotic signaling. Analysis of enzyme activity revealed that palmitate increased the activity of DPP4 nearly 2 folds, while F2 especially inhibited the activation. In addition, AMPK/mTOR, PI3K and mitochondrial pathways were regulated by AE, and this attenuated the palmitate-induced signaling cascades. In conclusion, AE is useful to prevent the exacerbation of β cell apoptosis, and it could potentially be used as adjuvant or nutraceutical therapy for diabetes.

## Introduction

Diabetes mellitus is a highly prevalent disease worldwide that is associated with significant morbidity and mortality [1]. Type 2 diabetes (T2DM), the most prevalent type of diabetes, is usually accompanied with obesity and characterized by insulin resistance [2]. Along with the pathogenesis, insulin signaling cascades have been shown to be altered in patients with T2DM [2]. Long-term exposure to diets high in glucose and fatty acids or over consumption of dietary sugar and fats has been reported to induce β cell apoptosis, consequently leading to the exacerbation of T2DM [3]. Lipotoxicity is considered to be an underlying mechanism of T2DM [4]. Recently, dipeptidyl peptidase-4 (DPP-4, a serine protease) inhibitors have emerged as useful tools for the treatment of T2DM. The mechanism of action relies on inhibiting the degradation of type 1 glucagon-like peptide (GLP-1), an incretin with a short half-life that specifically stimulates glucose-dependent insulin secretion from β cells and also effectively suppresses glucagon release from α cells [5]. Clinically, GLP-1 receptor (GLP-1R) agonists have been shown to efficiently protect β cells through various pathways, which include improving β-cell function, increasing β-cell mass, and enhancing an anti-apoptotic effect [4].

Accumulating evidence has demonstrated that the mechanism of one GLP-1 analogue, liraglutide, is mediated, at least in part, *via* the AMPK/mTOR signaling pathway, thereby preventing glucolipotoxicity in β cells [6]. In addition, exendin-4, a GLP-1R agonist, has been shown to be capable of preventing the palmitate-induced apoptosis of β cells by activating the PI3K/PKB pathway and inhibiting the mitochondrial pathway by downregulating Bax protein [4]. β cell apoptosis is considered to be a major cause of loss of β cells in diabetes [7]. Moreover, exendin (9–39), a GLP-1R antagonist, has been shown to inhibit the ameliorating effect of geniposide on palmitate-induced β cell apoptosis and active caspase-3 expression in INS-1 cells [7].

*Abelmoschus esculentus* (AE; commonly known as okra) is a flowering plant of the mallow family. AE fruits are popularly consumed as vegetables in many countries, and it is valued for its edible green seedpods [8]. AE is also used in folklore medicine, particularly for its anti-hyperglycemic effect in diabetes [9]. However, many cell and animal experiments have shown that the substantial mucilage content of AE makes it very difficult to isolate, analyze and further test its active components. Previously, we successfully isolated several subfractions (F1, F2, F3-F5, and FR) from AE using a series of successive extraction steps. Among these subfractions, F1 and F2 were found to be especially effective in suppressing the activity of DPP-4 [10]. This then successfully attenuated diabetic nephropathy, strongly suggesting that AE could serve as a promising adjuvant therapy for diabetes [10].

To date, no studies have investigated preventive strategies against the palmitate-induced apoptosis of β cells. Therefore, the aim of this study was to investigate whether subfractions F1 and F2 of AE could prevent palmitate-induced β cell apoptosis and which putative signals were involved.

## Materials and methods

### Chemicals

Antibodies to caspase 3, Bax, AMPK, pAMPK and mTOR were purchased from Santa Cruz Biotechnology (Santa Cruz, CA, USA). Antibodies to GLP-1R and DPP4 were provided by Abcam (Cambridge, UK), and antibodies to PI3K and pPI3K were products of from Cell Signaling (Danvers, MA, USA).

### Preparation of AE subfractions and chemical analysis

**Fig 1:**
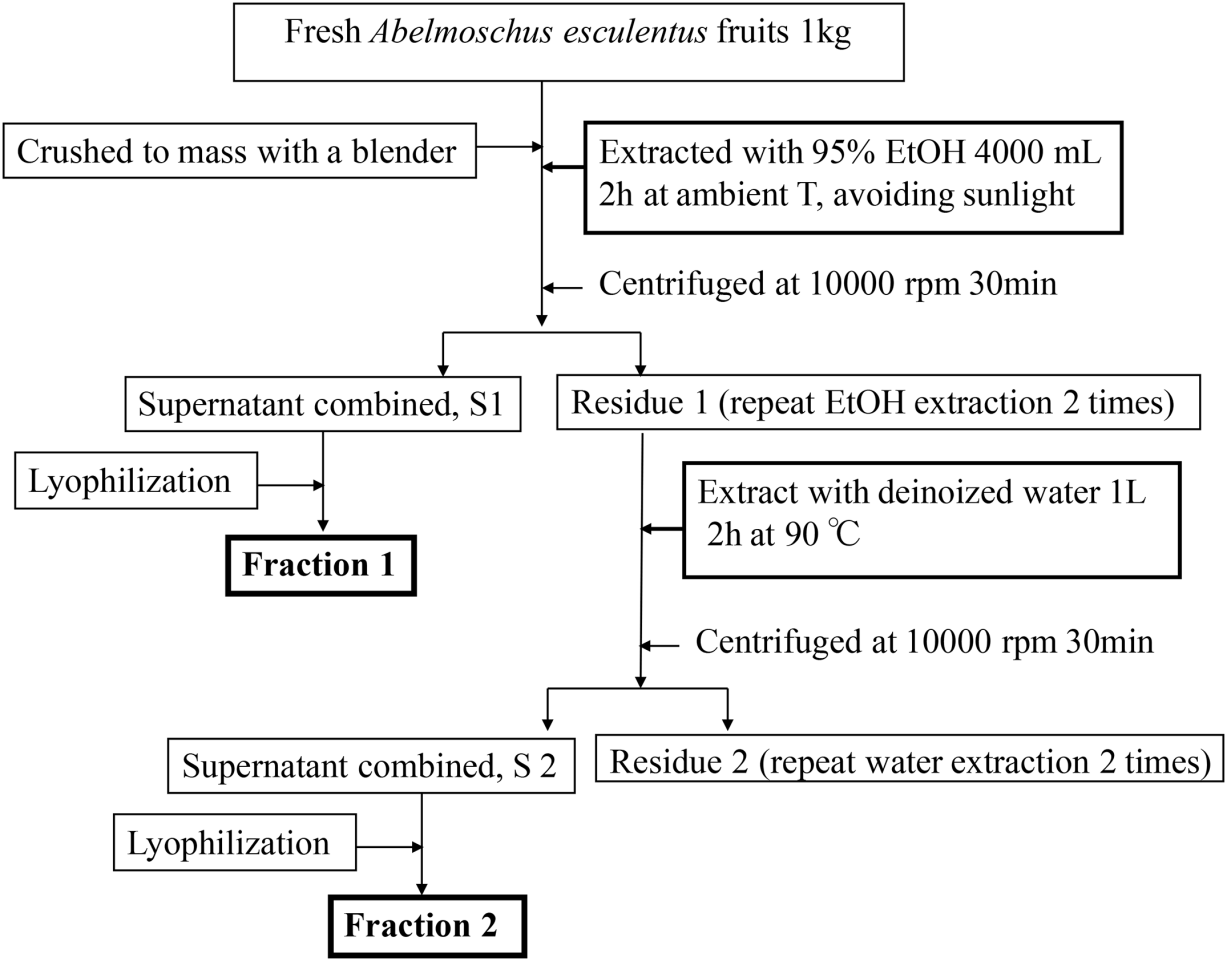
Procedures of extracting AE subfractions.

AE was purchased from Chuchi, Chiayi. The F1 and F2 subfractions of AE were prepared according to procedures shown in Fig 1. We previously reported LC-MS/MS analysis of F1, the alcohol-extracted fraction of AE [10]. F1 is comprised of at least 10 compounds, including quercetin glucosides (4.901 mg/g DW) and pentacyclic triterpene ester (4.301 mg/g DW) [S1 Table]. The F2 subfraction of AE contains a large amount of carbohydrates and polysaccharides [10]. In addition, monosaccharide and uronic acid analyses revealed that F2 is rich in uronic acid (23.14%), galactose (18.92%), glucose (18.26%) and myo-inositol (14.21%), and also rhamnose, glucosamine, and fucose [S2 Table]. The mean molecular weight of F2 was estimated to be 671 kDa in GPC analysis.

### Cell culture

The RINm5F cell line was purchased from the Bioresource Collection and Research Center (BCRC) of the Food Industry Research and Development Institute in Taiwan (BCRC no: 60410). The RINm5F cells were cultured in RPMI 1640 with 2 mM L-glutamine, 1.5 g/L sodium bicarbonate, 4.5 g/L glucose, 10 mM HEPES, 1.0 mM sodium pyruvate, and 10% fetal bovine serum. The cells were grown in a humidified incubator at 37°C in an atmosphere of 5% CO_2_ and 95% air, and then cells were inoculated at a density of 5 × 10^5^ cells per mL into a 6-cm dish for 24 h followed by treatment with or without various concentrations of palmitate and AE for 24 h. The cells were then harvested for the following experiments.

### MTT assay

The RINm5F cells were seeded at a density of 1 × 10^6^ cells per mL in a 24-well plate and incubated with each AE fraction and palmitate at various concentrations for 24 h. Thereafter, the medium was changed and the cells were incubated with 3-(4, 5-dimethylthiazol-2-yl)-2,5diphenyltetrazolium bromide (MTT, 0.5 mg/mL) for 4 h. The viable cells were directly proportional to the production of formazan. Following dissolution in isopropanol, the optical density was read at 563 nm with a spectrophotometer (Hitachi, U-3210) [10].

### Flow cytometry

Cell cycle distribution was determined using a FACScan system (Becton Dickinson Immunocytometry Systems, UK). Briefly, cell pellets were suspended in 0.25 mL trypsin buffer for 10 min at 25°C, after which 200 μL of trypsin inhibitor and RNase buffer were added to the suspension followed by incubation at 25°C for 10 min. Before the samples were analyzed, DNA was stained with 200 μL of cold (4°C) propidium iodine (PI) for 10 min in the dark on ice. PI was excited at 488 nm and the fluorescence signals were logarithmically amplified. The distribution of DNA content was expressed as sub-G1, G1, S, and G2/M. The percentage of sub-G1 cells was presented as the percent of apoptosis.

### Western blot

Cells were harvested into lysis buffer containing 50 mM TrisHCl (pH 6.8), 10% glycerol, 2% SDS, and 5% mercaptoethanol and then lysed by sonication. The cell lysate was centrifuged at 9300×g for 20 min at 4°C, and the supernatant was collected as the protein sample. After quantification, equal amounts of protein samples (50 μg) were subjected to 10% SDS-PAGE electrophoresis and transferred to nitrocellulose membranes (Millipore, Bedford, MA, USA). The membranes were blocked with 5% nonfat milk powder with 0.1% Tween-20 in TBS and then incubated at 4°C overnight with the primary antibodies to GLP-1R (1:1000), DPP-4 (1:1000), caspase 3 (1:1000), PI3K (1:1000), pPI3K (p85, Tyr 458) (1:500), Bax (1:1000), AMPK (1:1000), pAMPK (1:1000) and mTOR (1:1000). The membranes were then washed three times with 0.1% Tween-20 in TBS and incubated with the secondary antibodies (1:5000) conjugated to horseradish peroxidase (GE Healthcare, Little Chalfont, Buckinghamshire, UK). Band detection was performed using enhanced chemiluminescence using ECL Western blot detection reagents and exposure to FUJFILM Las-3000 (Tokyo, Japan). The protein quantity was determined by densitometry using FUJFILM-Multi Gauge V2.2 software.

### DPP-4 activity

Cells were seeded onto a 96-well plate at a density of 1 × 10^6^ per well. After treatment under various conditions as indicated, the cells in each well were lysed with 100 μL of NP-40 lysis buffer (containing 10 mM HEPES (pH 7.5), 142.5 mM KCl, 5 mM MgCl_2_, 1 mM EGTA, and 0.2% NP-40) and centrifuged at 9300×g for 20 min at 4°C. The supernatants were collected, and the protein concentrations were determined using the Bradford method. DPP-4 activity was measured using a DPP4/CD26 assay kit for biological samples (Enzo Life Sciences). Briefly, H-Gly–Pro-pNA, a chromogenic substrate of DPP-4, was hydrolyzed into dipeptide Gly-Pro and 4-nitroaniline. The rates of their appearance were measured spectrophotometrically at 405 nm. The activity was normalized to protein concentration, and then leveled in proportion to the controls.

### Statistical analysis

The statistical software package SPSS version 12.0 was used to analyze all data. One-way ANOVA was performed, and Bonferroni’s multiple comparisons were used for post-test analysis. A p value < 0.05 was taken to indicate statistical significance.

## Results

### Determination of the dose range feasible for experiments

**Fig 2:**
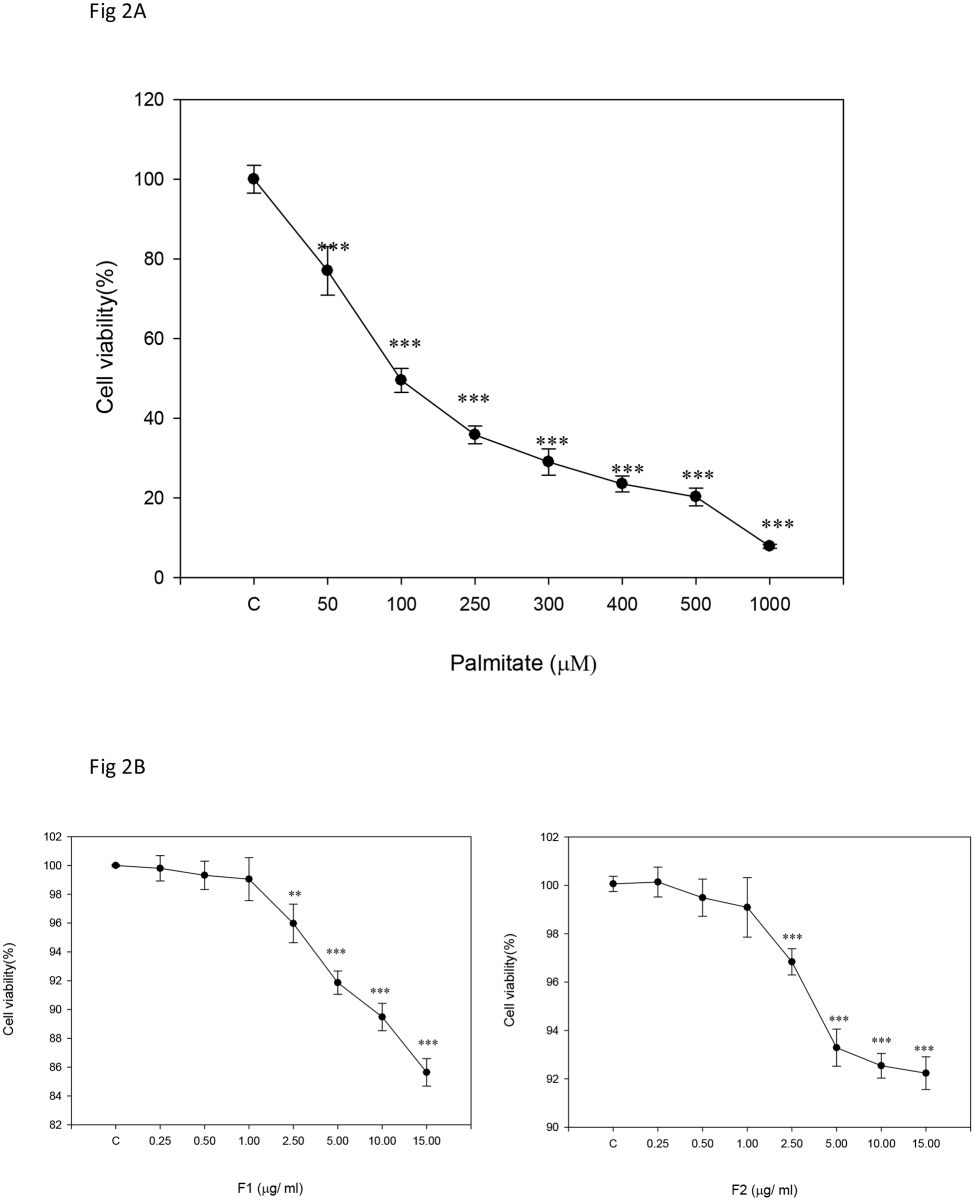
Cytotoxicity test for palmitate and AE subfractions. β cells were incubated for 24 h with or without different concentrations of (A) palmitate, (B) F1 and F2. Cell viability was calculated as percentage compared to the control group. Data are presented as means ± SD (n = 3) and analyzed using ANOVA. *p < 0.05, **p < 0.01, ***p < 0.001, compared to the controls.

Fig 2A shows that palmitate at a dose above 50 μM significantly inhibited cell viability. The IC_50_ of palmitate was estimated to be 100 μM (Fig 2A), which was thus used in all of the experiments. In contrast, at a dose below 1 μg/mL, cell viability did not seem to be altered very much by F1 or F2, and hence this dose was defined as the maximum concentration to be used for the β cells (Fig 2B). In the following experiments, two doses of F1 and F2 were used, i.e. 0.25 μg/mL and 1 μg/mL, which were classified as L and H, respectively.

### AE subfractions prevented palmitate-induced β cell apoptosis

**Fig 3:**
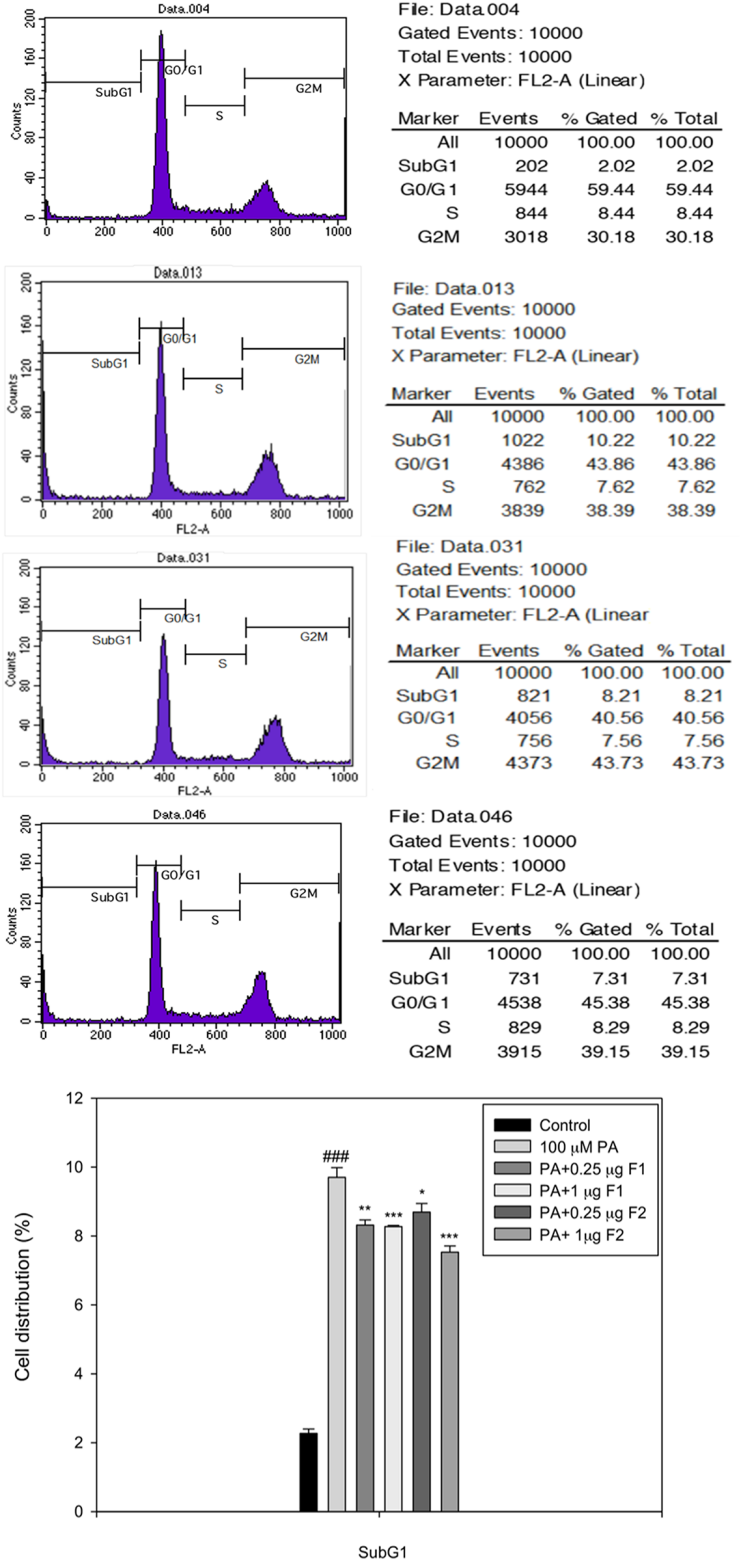
Effect of AE on palmitate-induced apoptosis. β cells were incubated for 24 h with or without palmitate and AE treatment, and then analyzed using flow cytometry. Top to bottom indicates the controls, palmitate, palmitate with 1 μg/mL of F1, and palmitate with 1 μg/mL of F2, respectively, with the percentage of cell distribution. The percentage of sub-G1 was calculated. Data are presented as means ± SD (n = 3) and analyzed using ANOVA. ###p < 0.001, compared to the controls. *p < 0.05, **p < 0.01, ***p < 0.001, compared to palmitate treatment.

**Fig 4:**
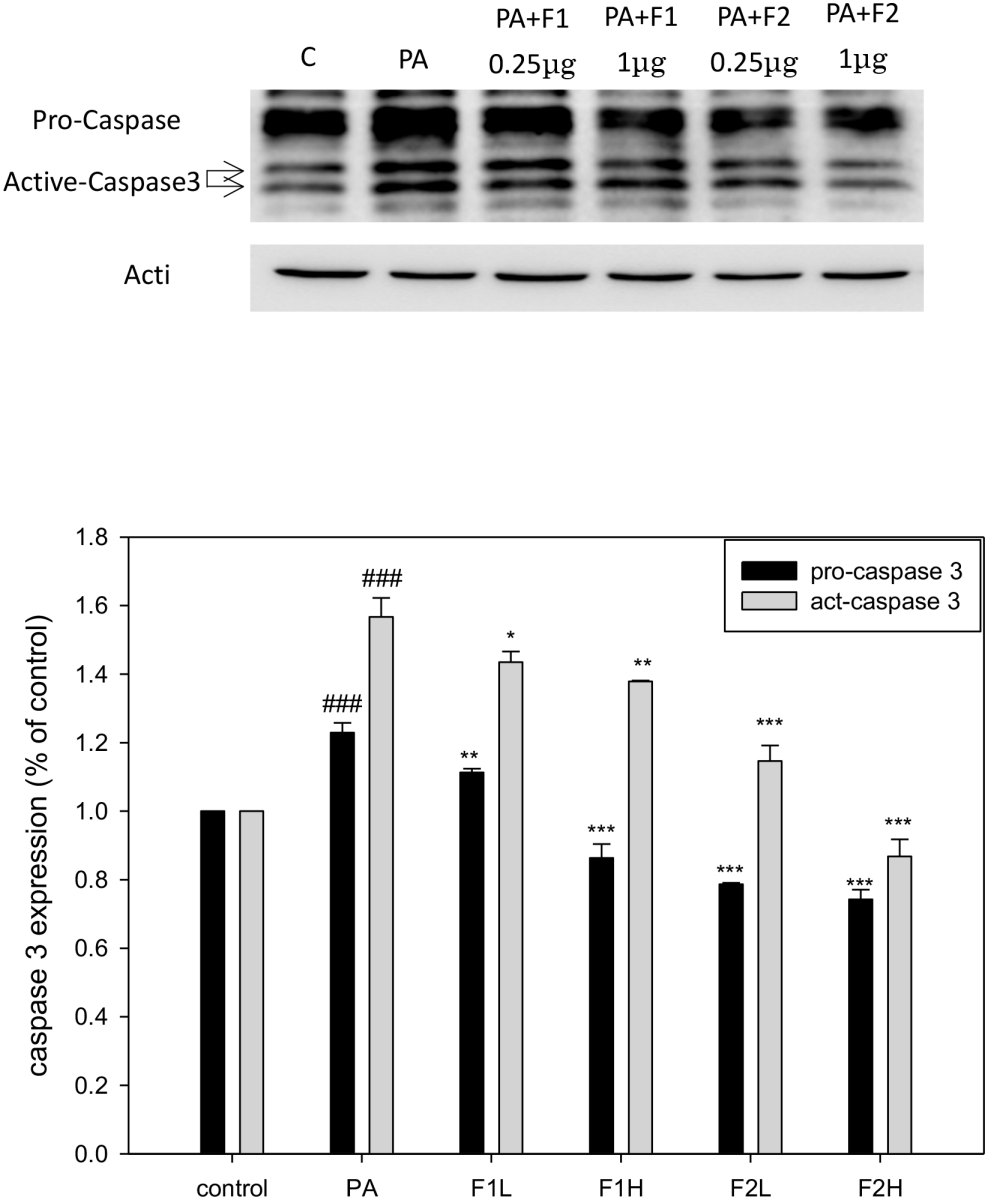
Effect of AE on palmitate-induced activation of caspase 3. β cells were incubated for 24 h with or without palmitate and AE treatment, and then analyzed using Western blot. The protein levels of pro-caspase and active caspase were calculated as a percentage compared to that of the control group. Data are presented as means ± SD (n = 3) and analyzed using ANOVA. ###p < 0.001, compared to the controls. *p < 0.05, **p < 0.01, ***p < 0.001, compared to palmitate treatment.

Cells treated with different conditions were analyzed using flow cytometry. Apoptosis was defined as cells distributed at the sub G1 stage. Fig 3 shows that palmitate induced a five-fold increase in sub G1 cell population compared to the controls (10.22% vs. 2.02%). Treatment with F1H or F2H reduced the percentage of sub G1 to 8.21% and 7.31%, respectively. In addition, palmitate increased the expressions of pro-caspase 3 and active-caspase 3 by approximately 1.2 and 1.6 folds, respectively, compared to the controls. F1 effectively suppressed the expression of caspase, while F2H seemed to be more specifically effective in inhibiting pro-caspase and act-caspase. The latter were suppressed by 0.8 and 0.9 folds, respectively, which was far below that of the controls (Fig 4), suggesting that AE subfractions can potentially inhibit palmitate-induced β cell apoptosis.

### AE subfractions ameliorated the downregulation of GLP-1R and the upregulation of DPP-4 by palmitate

**Fig 5:**
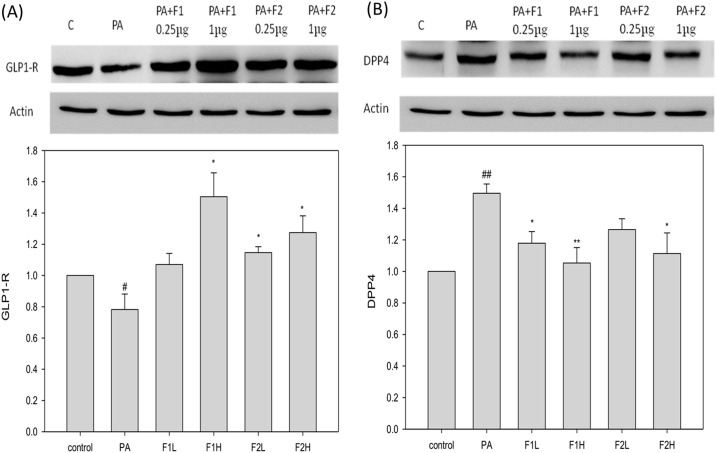
Effect of AE on the palmitate-induced decrease in GLP-1R and increase in DPP-4. β cells were incubated for 24 h with or without palmitate and AE treatment, and then analyzed using Western blot. The protein levels of (A) GLP-1R and (B) DPP-4 were calculated as a percentage compared to the control group. Data are presented as means ± SD (n = 3) and analyzed using ANOVA. #p < 0.05, ##p < 0.01, compared to the controls. *p < 0.05, **p < 0.01, compared to palmitate treatment.

As shown in Fig 5A, palmitate significantly downregulated GLP-1R. Treatment with F1 and F2, especially at a dose of 1 μg/mL, ameliorated the expression of GLP-1R. In contrast, palmitate upregulated DPP-4 by approximately 1.5 folds (Fig 5B), and treatment with F1 and F2, preferably at a higher dose, apparently restored the expression of DPP-4.

### AE subfractions ameliorated the upregulation of AMPK and Bax and downregulation of mTOR and PI3K induced by palmitate

**Fig 6:**
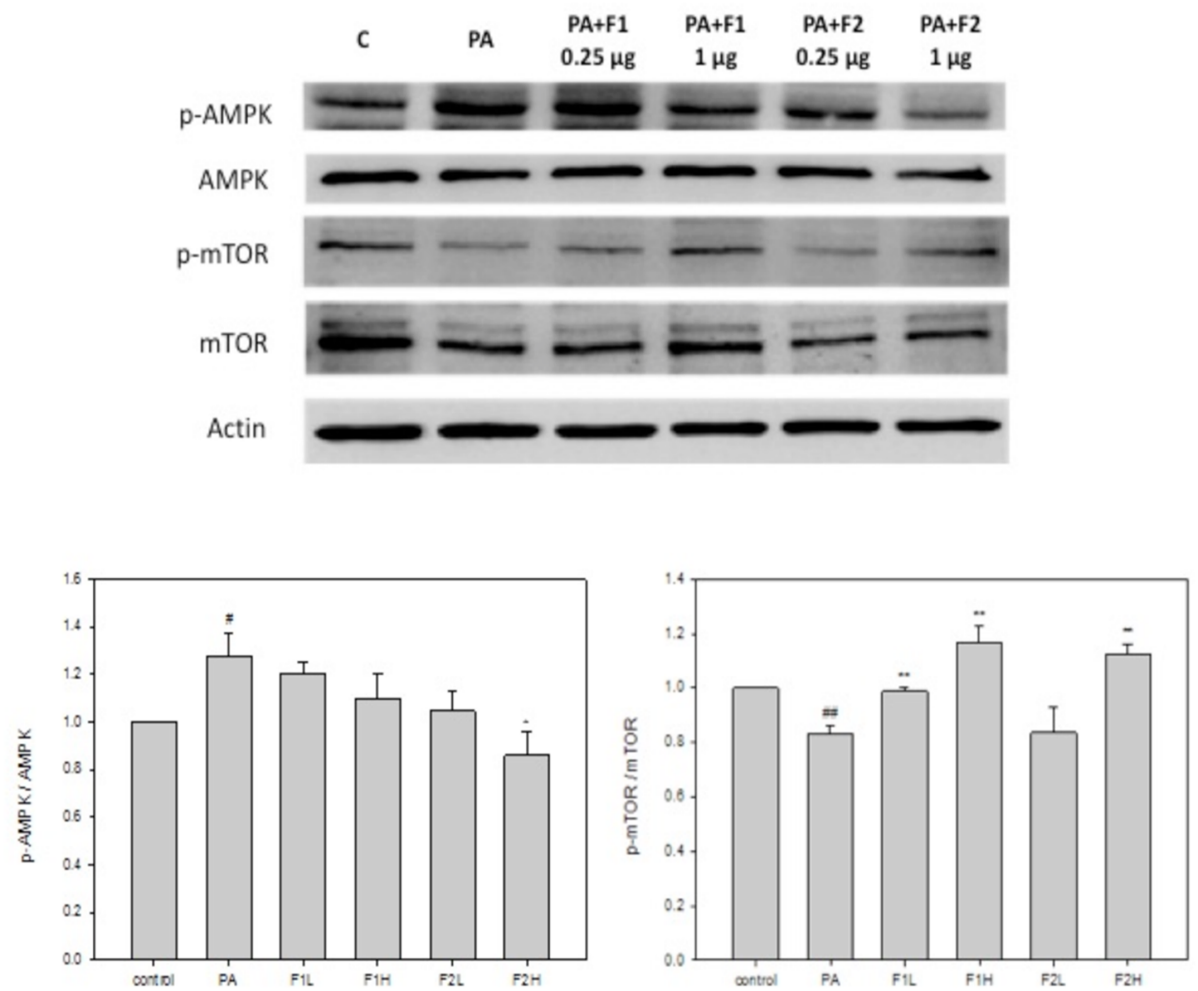
Effect of AE on palmitate-induced signals of AMPK and mTOR. β cells were incubated for 24 h with or without palmitate and AE treatment, and then analyzed using Western blot. The phosphorylated or total protein levels of AMPK and mTOR were calculated as a percentage compared to the control group. Data are presented as means ± SD (n = 3) and analyzed using ANOVA. #p < 0.05, ##p < 0.01, compared to the controls. *p < 0.05, **p < 0.01, compared to palmitate treatment.

**Fig 7:**
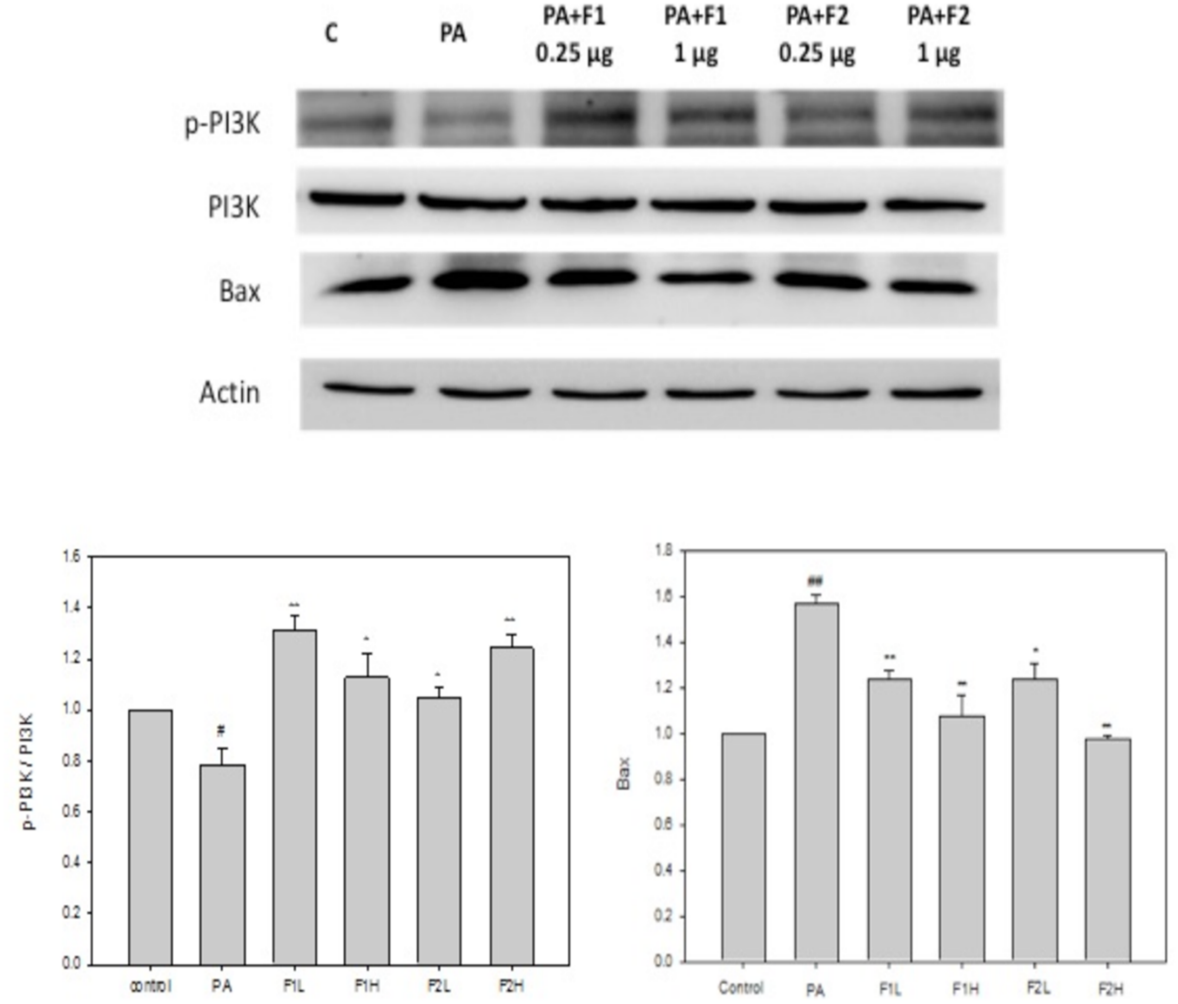
Effect of AE on palmitate-induced signals of PI3K and Bax. β cells were incubated for 24 h with or without palmitate and AE treatment, and then analyzed using Western blot. The phosphorylated or total protein levels of PI3K and Bax were calculated as a percentage compared to the control group. Data are presented as means ± SD (n = 3) and analyzed using ANOVA. #p < 0.05, ##p < 0.01, compared to the controls. *p < 0.05, **p < 0.01, compared to palmitate treatment.

Palmitate upregulated AMPK by approximately 1.3 folds compared to the controls. F1 seemed not to be as effective as F2 in ameliorating this effect (Fig 6). Conversely, palmitate downregulated mTOR, and both F1 and F2 significantly restored the level of mTOR (Fig 6). Fig 7 shows that palmitate downregulated PI3K by about 20%, and that treatment with F1 and F2 efficiently increased the level of PI3K. In addition, palmitate significantly upregulated Bax by 1.6 folds, and both F1 and F2, in particular at a higher dose, efficiently recovered the level of Bax (Fig 7). These results suggest that AE subfractions may be potentially effective in ameliorating the putative signaling pathways involved in palmitate-induced apoptosis.

### DPP4 mediated the palmitate-induced apoptotic cascades in β cells

**Fig 8:**
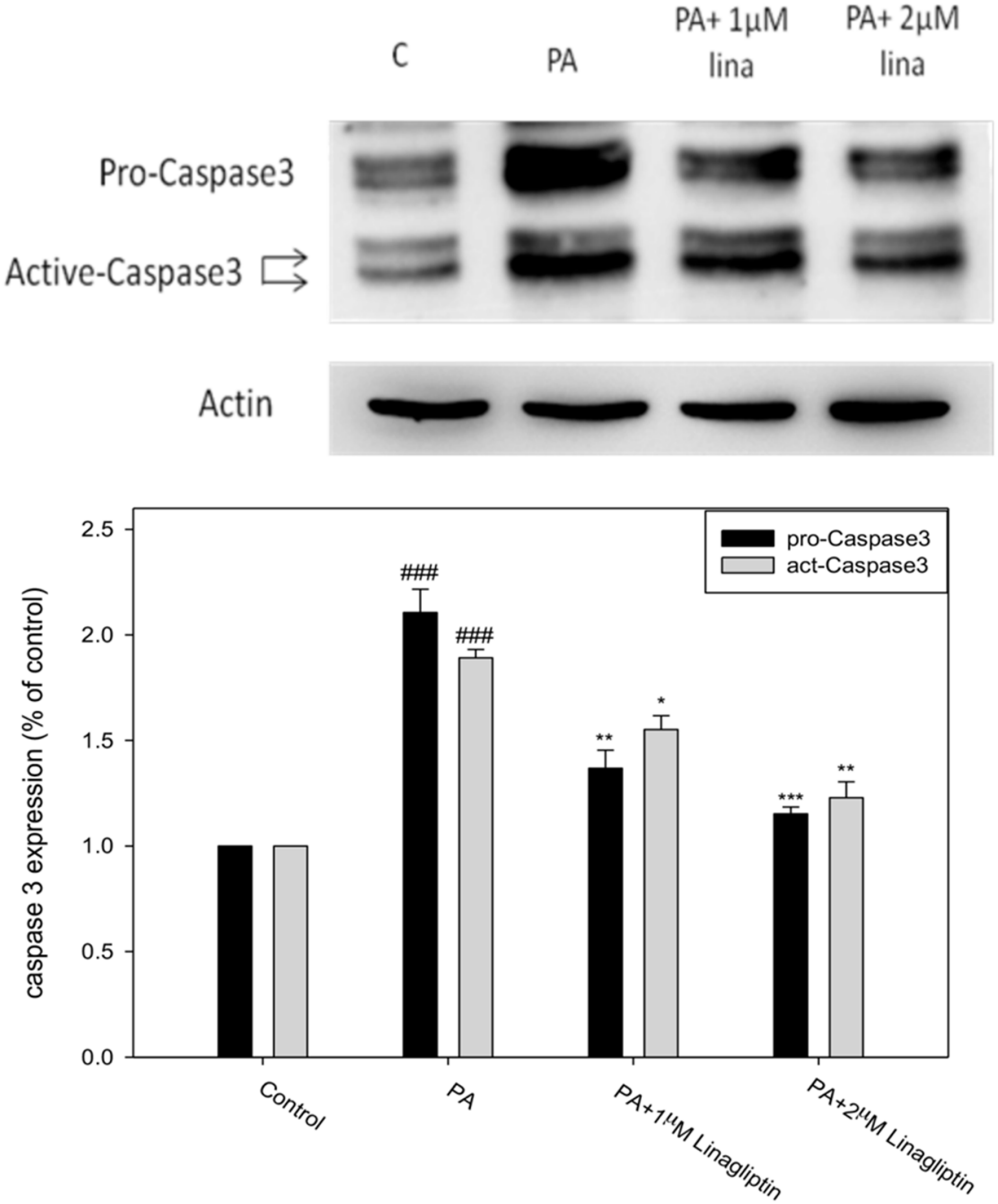
Effect of AE on DPP-4-mediated apoptosis. β cells were incubated for 24 h with or without palmitate and the DPP-4 inhibitor linagliptin. The protein levels of pro-caspase and active caspase were analyzed using Western blot and calculated as a percentage compared to the control group. Data are presented as means ± SD (n = 3) and analyzed using ANOVA. ###p < 0.001, compared to the controls. *p < 0.05, **p < 0.01, ***p < 0.001, compared to palmitate treatment.

**Fig 9:**
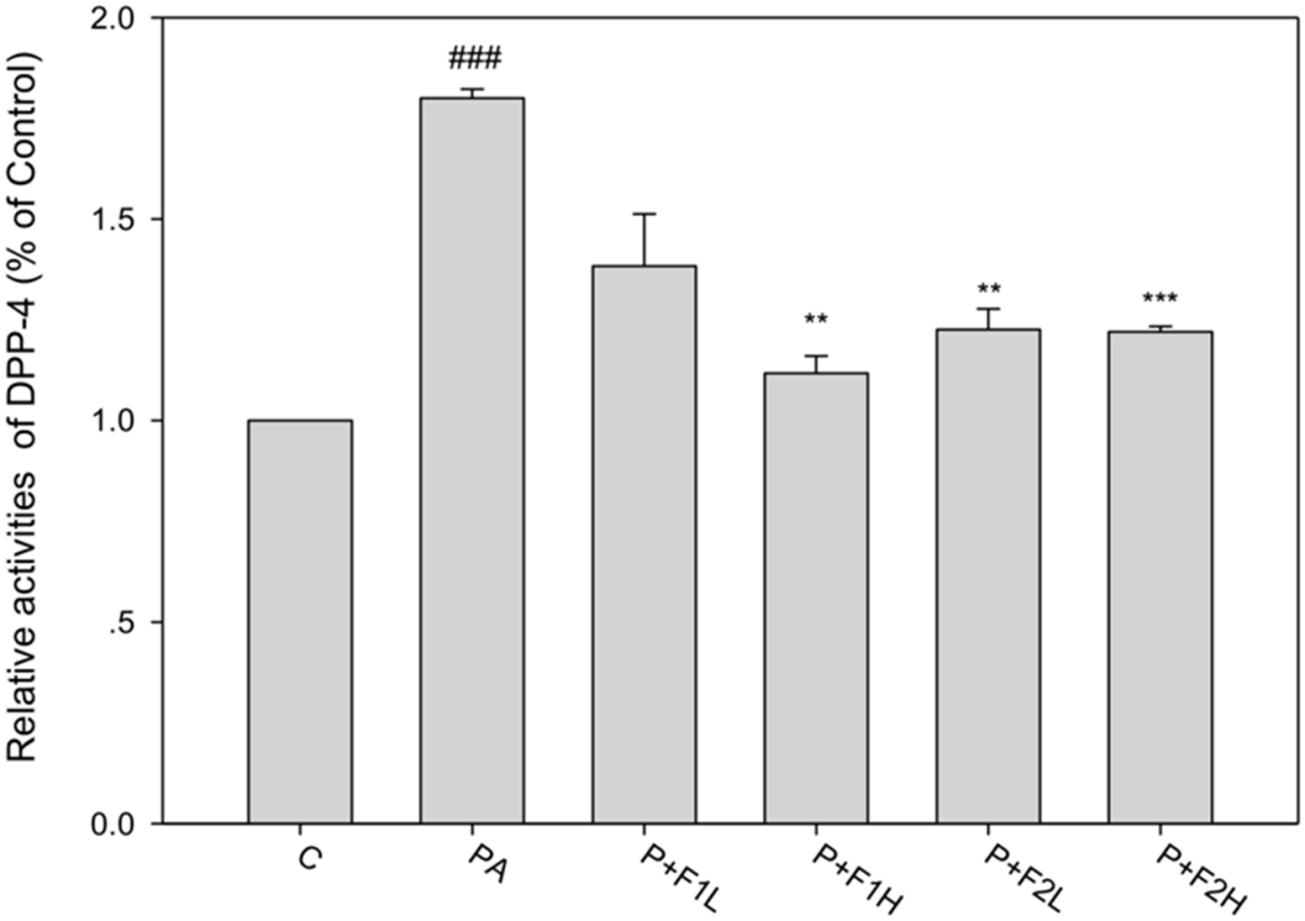
Effect of AE on DPP-4-mediated apoptosis. β cells were incubated for 24 h with or without palmitate and AE. DPP-4 activity was analyzed and calculated as a percentage compared to the control group. Data are presented as means ± SD (n = 3) and analyzed using ANOVA. ###p < 0.001, compared to the controls. **p < 0.01, ***p < 0.001, compared to palmitate treatment.

To investigate whether GLP-1R/DPP-4 mediated apoptotic signaling in β cells, the GLP-1R antagonist exendin (9–39) and DPP-4 inhibitor linagliptin were used to interfere with the function of GLP-1R and DPP-4, respectively. Fig 8 shows that linagliptin attenuated the expression of caspase 3 (the appropriate dose range of linagliptin had previously been assessed using MTT, and its effect had been analyzed against the activity of DPP-4), suggesting that DPP-4 is involved in apoptotic signaling. Fig 9 shows that palmitate stimulated DPP-4 activity by almost 2 folds, whereas F1H decreased the activation. Treatment with F2, even at a dose as low as 0.25 μg/mL, significantly suppressed the activation of DPP-4, suggesting that AE suppressed apoptotic signaling via the regulation of DPP-4. However, exendin (9–39) completely failed to alter the expression of caspase 3 in the AE-treated groups, suggesting that GLP-1R is not involved in either the F1 or F2 rescue process.

## Discussion

In the present study, we demonstrated that AE subfractions are effective in preventing palmitate-induced apoptosis of β cells, and that the mechanism of this effect appears to be via inhibiting the activity of DPP-4. In addition, the AMPK/mTOR, PI3K and mitochondrial pathways were regulated by AE, and this could attenuate palmitate-induced signal cascades. Of note, the subfractions F1 and F2 were efficacious at a dose as low as 1 μg/mL, but the IC_50_ of F1 and F2 was estimated as high as 60 and 96 μg/mL, respectively. These results suggested AE subfractions have promising quality and bioactivity with applicable dose range leads no cytotoxicity. AE subfractions are feasible to be used clinically.

By measuring the amount of dye taken up by the cell and indirectly the amount of DNA, subG1 arrest is deemed as the late phase of apoptosis which is rich of 180-200bp DNA fragments cleaved by endonucleases. As well known, the subsequent phase G1 is transcription associated during the cell cycle. In view of the molecular cytology of apoptosis, the transcription of some survival proteins must be impeded by loss of the DNA content.

Although apoptosis was inhibited by both F1 and F2, F2H seemed to be superior in modulating the activation of AMPK and DPP-4. Our recent in vivo investigation showed that F2 is the most effective subfraction with respect to the prevention of hyperglycemia, suppression of HbA1C, and improvement of insulin sensitivity (undergoing review). As well, F2 attenuated the β islet destruction in type 2 diabetic rats [S1 Fig]. The present study apparently provides further evidence that these in vivo effects of AE can be attributed to its ability to prevent the apoptosis of β cells, thus maintaining the secretion and function of insulin.

The inhibition of DPP-4 has been shown to protect against β cell apoptosis, restore β cell mass, and normalize islet morphology in Gck(+/-) mice fed with sucrose and linoleic acid [3]. It has also been suggested that chronic endoplasmic reticulum (ER) stress can trigger β cell apoptosis. Treatment with vildagliptin has been shown to promote β cell survival in db/db mice, and that this was associated with down-regulated markers of ER stress including TRIB3, ATF-4 and CHOP [11]. Gemigliptin has been shown to effectively inhibit ER stress-induced apoptosis and inflammation in cardiomyocytes via Akt/PERK/CHOP and IRE1alpha/JNK-p38 pathways [12]. In addition, sitagliptin has been shown to attenuate hypoxia-induced apoptosis and autophagy of mesenchymal stem cells [13]. Although the inhibition of DPP-4 in vivo should extend the half-life of GLP-1, the in vitro findings suggest that DPP-4 per se is involved in the apoptotic signal cascades. Of note, the AE subfractions potentially upregulated GLP-1R. Although the anti-apoptotic effect was not mediated by the receptor, the increased expression of GLP-1R may benefit the conjugation of GLP-1 to the receptor in vivo, leading to a more efficient protection of β cells.

AMPK is an energy sensor that regulates cellular metabolism. With chronic high glucose treatment, AMPK activation potentiates β-cell apoptosis through enhanced reactive oxygen spices production and downregulation of GCK [14]. Liraglutide has been shown to increase β-cell viability, however this effect has been shown to be abated by several pathway blockers: the AMPK activator AICAR and the mTOR inhibitor rapamycin [6]. It has also been suggested that AMPK activation mediates the downstream mitochondrial-dependent and ER stress-triggered apoptotic pathways [15]. However, several previous studies have shown that AMPK regulates SREBP, thus reducing downstream lipogenesis and enhancing lipolysis. Hibiscus sabdariffa polyphenols have been shown to decrease hepatic lipid content by activating AMPK and reducing SREBP-1, thus inhibiting the expression of fatty acid synthase and HMG-CoA reductase [16]. In addition, alpha-lipoic acid has been shown to increase AMPK phosphorylation in liver cells, thus preventing the insulin-stimulated release of SREBP-1c and the development of non-alcoholic fatty liver disease [17]. Furthermore, the activation of AMPK has been shown to increase liver fatty acid oxidation and the exocytosis of lipoprotein [18]. While palmitate-induced apoptosis has been shown to be accompanied with the activation of AMPK, it is possible that an increase in AMPK activity may be required for fatty acid-induced fatty acid oxidation and prevention of lipotoxicity [19].

The F2 subfraction of AE contains a large amount of carbohydrates and polysaccharides. Monosaccharide and uronic analyses have revealed that F2 is enriched with uronic acid, galactose, glucose and myo-inositol, as well as rhamnose, fucose, and glucosamine [10]. Fucose-rich oligo- and polysaccharides can stimulate cell proliferation and promote cell survival, thus slowing the aging of skin fibroblasts [20]. In addition, the O-linked attachment of N-acetyl-glucosamine (O-GlcNAc) has been shown to modify DNA binding, enzyme activity, protein-protein interactions, the half-life of proteins, and subcellular localization. Although controversial, O-GlcNAc could play a role in the regulation of cell survival [21].

The F1 subfraction of AE is composed of at least 10 compounds, including quercetin and its glucosides. Quercetin has been reported to attenuate cell apoptosis in focal cerebral ischemia via activation of the BDNF-TrkB-PI3K/Akt signaling pathway [22]. In addition, quercetin has been shown to block JNK- and p38 MAPK-related signaling triggered by oxidation, and it may regulate the expression of apoptotic downstream genes, thereby preventing apoptosis and promoting cell survival [23]. Quercetin 3-O-methyl ether has been shown to protect FL83B liver cells from Cu (2+)-induced apoptosis and mitochondrial dysfunction, and PI3K, Akt and Erk have been shown to be critically involved in the survival of such quercetin ether-treated cells [24]. We hypothesize that the regulation of PI3K and Bax by the F1 fraction may be due to its quercetin derivatives.

In conclusion, AE may be useful in preventing exacerbation of β cell apoptosis in patients with T2DM. Our findings indicate that AE deserves further research with regards to the development of an adjuvant or nutraceutical therapy for T2DM.

## Supporting information

S1 Table Identified compounds in F1. (DOC)

S2 Table Monosaccharides in F2. (DOC)

S1 Fig Effect of F2 on β islet cells of type 2 diabetic rats (top to bottom: control, type 2 diabetic rats, diabetic rats fed with 0.45 mg/kg BW of F2). (DOC)
