# Supplementary material for: Active subfractions of Abelmoschus esculentus substantially prevent free fatty acid-induced β cell apoptosis via inhibiting dipeptidyl peptidase-4
Source: PLoS One. 2017 Jul 17;12(7):e0180285. doi: 10.1371/journal.pone.0180285 (PMC5513409; doi:10.1371/journal.pone.0180285)
Supplement: S1 Table — (DOC) [file pone.0180285.s001.doc]

**S1 Table. Identified compounds in F1**

| Peak No. | Compound | mg/g DW |
| --- | --- | --- |
| 1 | quercetin 3-O-glucosyl (1 →6)glucoside | 1.848 |
| 2 | quercetin 3-O-xylosyl (1 → 2) glucoside | 0.628 |
| 3 | quercetin 3-*O-*glucoside | 2.425 |
| 4 | Flavonoid glucoside | 9.426 |
| 5 | Flavonoid glucoside | 0.406 |
| 6 | Unknown | 0.285 |
| 7 | Unknown | 0.568 |
| 8 | Unknown | 1.142 |
| 9 | Pentacyclic triterpene ester | 3.798 |
| 10 | Pentacyclic triterpene ester | 0.503 |
