# Supplementary material for: Active subfractions of Abelmoschus esculentus substantially prevent free fatty acid-induced β cell apoptosis via inhibiting dipeptidyl peptidase-4
Source: PLoS One. 2017 Jul 17;12(7):e0180285. doi: 10.1371/journal.pone.0180285 (PMC5513409; doi:10.1371/journal.pone.0180285)
Supplement: S2 Table — (DOC) [file pone.0180285.s002.doc]

**S2 Table. Monosaccharides in F2**

| Sugar | Wt % |
| --- | --- |
| rhamnose | 9.79 |
| fucose | 7.30 |
| mannose | 0.43 |
| glucosamine | 8.86 |
| myo-inositol | 14.21 |
| glucose | 18.26 |
| galactose | 18.92 |
| uronic acid (galacturonic acid 16.26%) | 23.14 |
