# Supplementary material for: Active subfractions of Abelmoschus esculentus substantially prevent free fatty acid-induced β cell apoptosis via inhibiting dipeptidyl peptidase-4
Source: PLoS One. 2017 Jul 17;12(7):e0180285. doi: 10.1371/journal.pone.0180285 (PMC5513409; doi:10.1371/journal.pone.0180285)

**S1 Fig. Effect of F2 on beta islet of type 2 diabetic rats (top to bottom: control, type 2 diabetic rats, diabetic rats fed with 0.45 mg/kg BW of F2)**


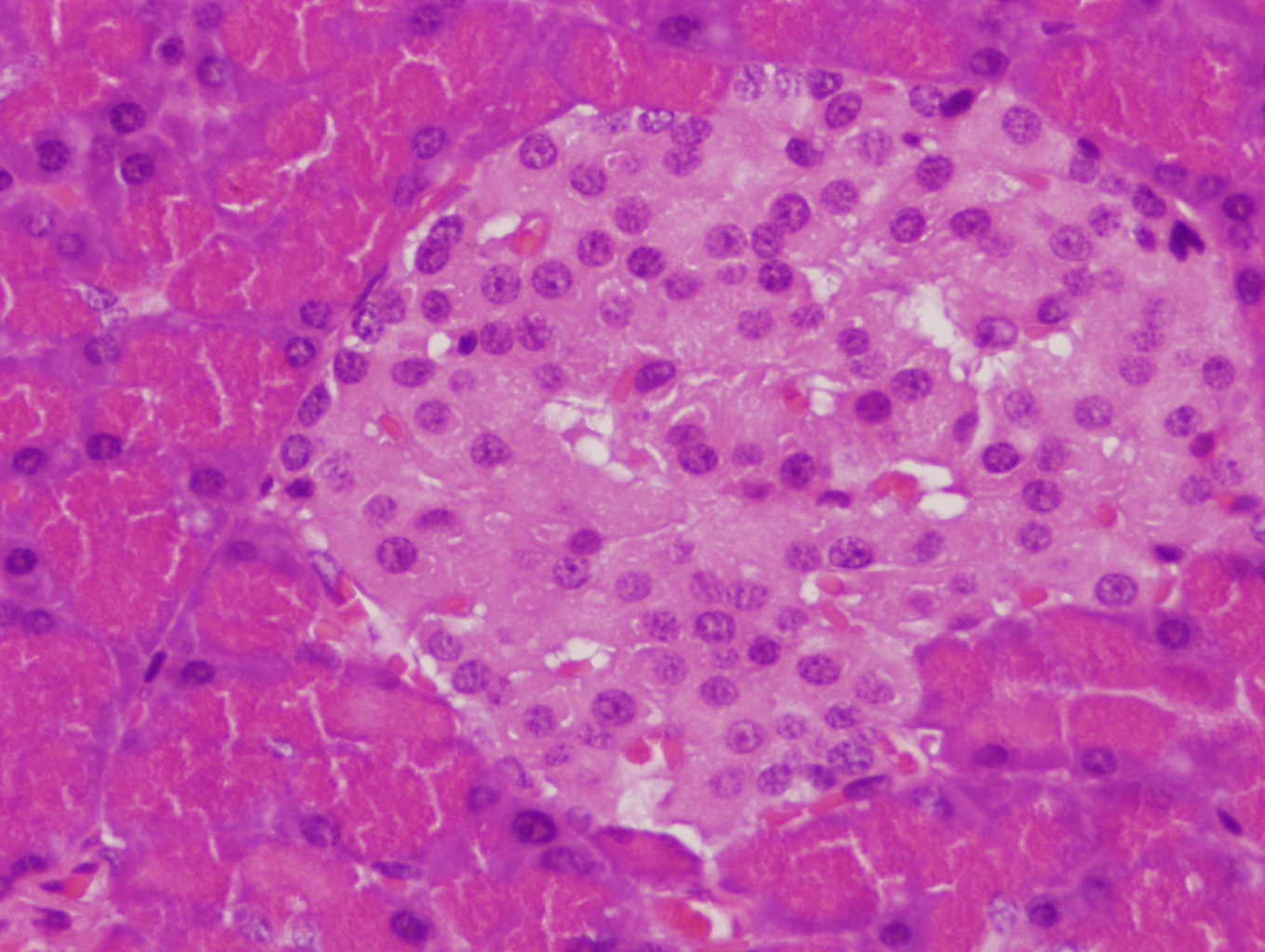


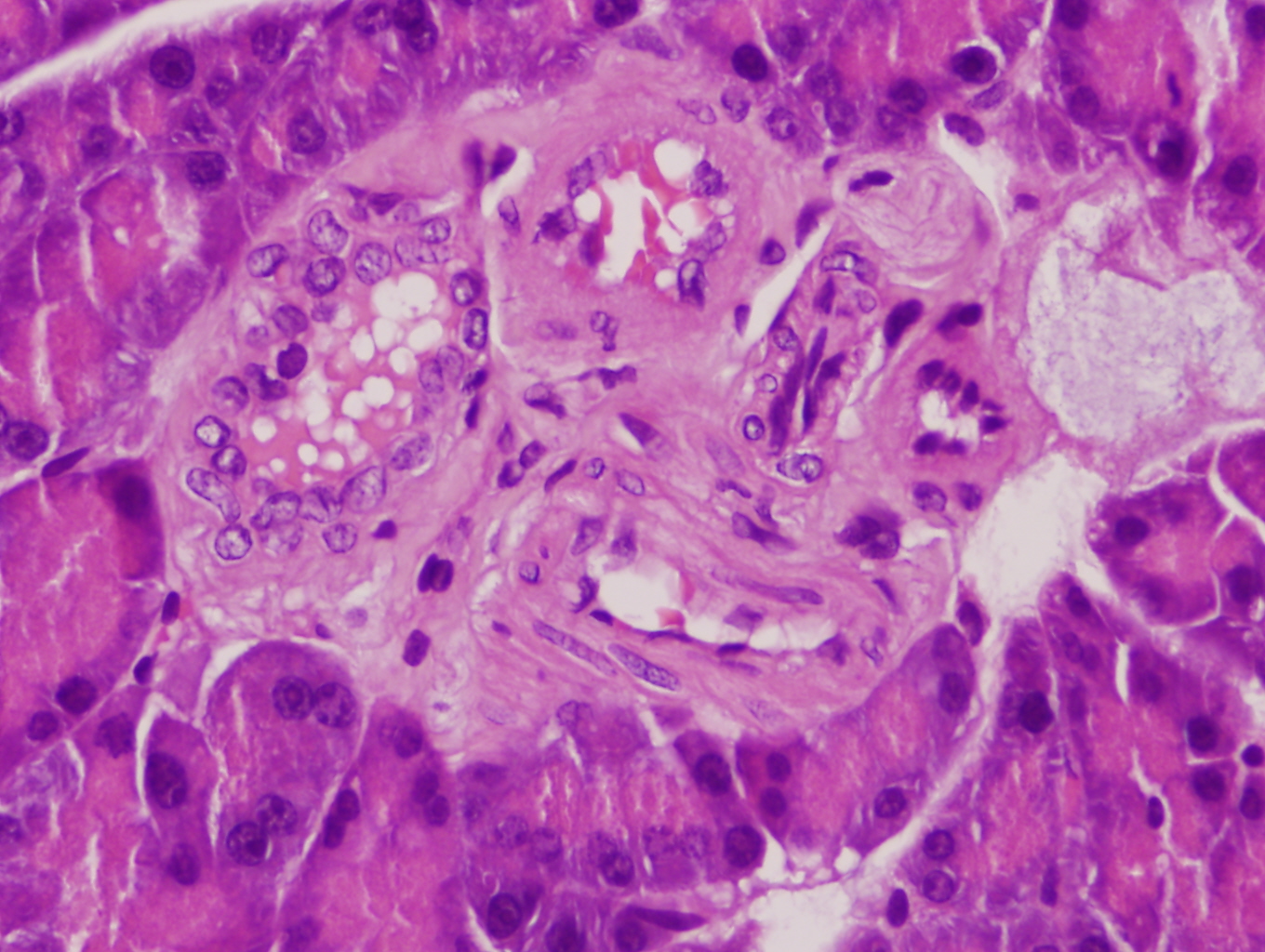


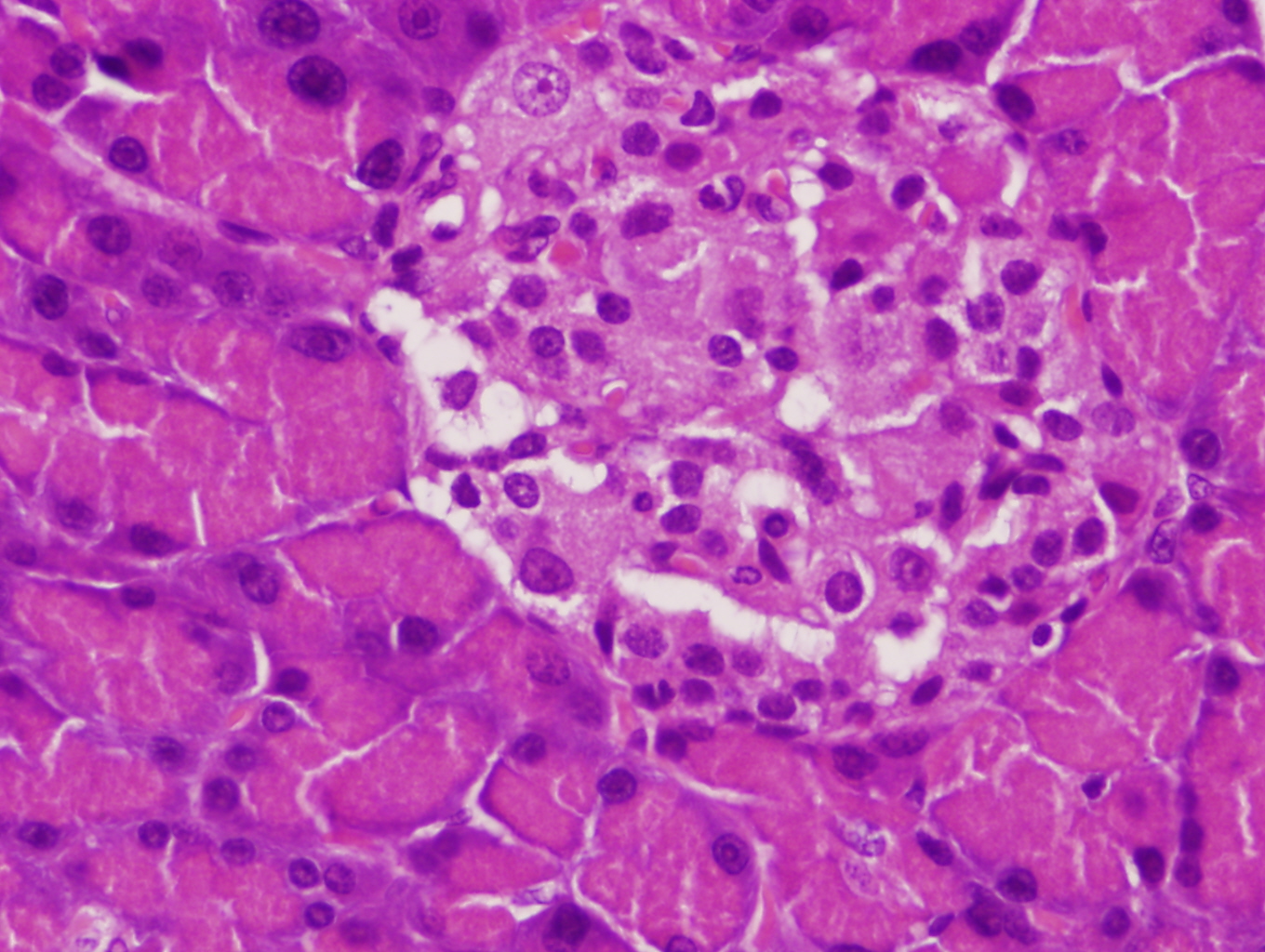

Supplement: S1 Fig — (DOC) [file pone.0180285.s003.doc]
